# Culture and Immunomodulation of Equine Muscle-Derived Mesenchymal Stromal Cells: A Comparative Study of Innovative 2D versus 3D Models Using Equine Platelet Lysate

**DOI:** 10.3390/cells13151290

**Published:** 2024-07-31

**Authors:** J. Duysens, H. Graide, A. Niesten, A. Mouithys-Mickalad, G. Deby-Dupont, T. Franck, J. Ceusters, D. Serteyn

**Affiliations:** 1 Revatis SA, Rue de la Science 8, 6900 Marche-En-Famenne, Belgium; helene.graide@revatis.com (H.G.); justine.ceusters@revatis.com (J.C.); didier.serteyn@uliege.be (D.S.); 2 Centre of Oxygen Research and Development (CORD), University of Liege, 4000 Liege, Belgium; ariane.niesten@uliege.be (A.N.); amouithys@uliege.be (A.M.-M.); fa511444@skynet.be (G.D.-D.); t.franck@uliege.be (T.F.)

**Keywords:** mesenchymal stromal cells, horse, 2D culture, 3D culture, platelet lysate

## Abstract

Muscle-derived mesenchymal stromal cells (mdMSCs) hold great promise in regenerative medicine due to their immunomodulatory properties, multipotent differentiation capacity and ease of collection. However, traditional in vitro expansion methods use fetal bovine serum (FBS) and have numerous limitations including ethical concerns, batch-to-batch variability, immunogenicity, xenogenic contamination and regulatory compliance issues. This study investigates the use of 10% equine platelet lysate (ePL) obtained by plasmapheresis as a substitute for FBS in the culture of mdMSCs in innovative 2D and 3D models. Using muscle microbiopsies as the primary cell source in both models showed promising results. Initial investigations indicated that small variations in heparin concentration in 2D cultures strongly influenced medium coagulation with an optimal proliferation observed at final heparin concentrations of 1.44 IU/mL. The two novel models investigated showed that expansion of mdMSCs is achievable. At the end of expansion, the 3D model revealed a higher total number of cells harvested (64.60 ± 5.32 million) compared to the 2D culture (57.20 ± 7.66 million). Trilineage differentiation assays confirmed the multipotency (osteoblasts, chondroblasts and adipocytes) of the mdMSCs generated in both models with no significant difference observed. Immunophenotyping confirmed the expression of the mesenchymal stem cell (MSC) markers CD-90 and CD-44, with low expression of CD-45 and MHCII markers for mdMSCs derived from the two models. The generated mdMSCs also had great immunomodulatory properties. Specific immunological extraction followed by enzymatic detection (SIEFED) analysis demonstrated that mdMSCs from both models inhibited myeloperoxidase (MPO) activity in a strong dose-dependent manner. Moreover, they were also able to reduce reactive oxygen species (ROS) activity, with mdMSCs from the 3D model showing significantly higher dose-dependent inhibition compared to the 2D model. These results highlighted for the first time the feasibility and efficacy of using 10% ePL for mdMSC expansion in novel 2D and 3D approaches and also that mdMSCs have strong immunomodulatory properties that can be exploited to advance the field of regenerative medicine and cell therapy instead of using FBS with all its drawbacks.

## 1. Introduction

Mesenchymal stromal cells (MSCs) have emerged as promising candidates for regenerative medicine due to their immunomodulatory properties and capacity to differentiate into various cell types [1,2]. In vitro expansion of MSCs is essential to obtain a sufficient number of cells for many applications including cell therapy, drug development and biomedical engineering. However, despite ethical concerns, fetal bovine serum (FBS) remains the supplement of choice in traditional culture methods to obtain large numbers of cells [3,4]. Indeed, over the past 20 years, the consumption of FBS, in the European Union only, has increased from approximately 500,000 to 800,000 L per year, which represents more than 1 million bovine fetuses killed to meet the culture needs [5,6,7]. In addition, FBS has numerous disadvantages including batch-to-batch variability, immunogenicity, xenogenic contamination and regulatory compliance issues. Moreover, several studies have shown that FBS may contain lipopolysaccharides, an endotoxin or xenogenic antigens and can lead to a change in the phenotype of the MSCs making these cells immunogenic for the recipient [8,9,10,11]. Nevertheless, the direction in cell culture suggests, as recommended by the European Medicines Agency (EMA) and the International Society for Cell and Gene Therapy (ISCT), a shift towards the use of xeno-free culture supplements, with blood products from the same species, and this result is the most promising solution [12,13].

Platelet lysate (PL) has attracted attention as an FBS substitute for MSC expansion due to its rich content of growth factors, cytokines and bioactive molecules that mimic the physiological microenvironment [14]. Equine platelet lysate (ePL), a derivative of platelet-rich plasma (PRP), can be easily obtained from equine blood by using a plasmapheresis device, providing scalable production and a reliable source of supplementation. Additionally, previous research has demonstrated that ePL serves as a viable substitute for fetal bovine serum in equine adipose-derived mesenchymal stromal cell (ad-MSC) culture without altering fundamental MSC characteristics [15]. Nevertheless, there is a lack of standardization in human and animal cell cultures using platelet lysate due to the various techniques employed to obtain a quality platelet lysate [15].

There are three critical parameters for culturing MSCs in 2D, including the method used for platelet lysis, the platelet concentration added to the basal medium and the final heparin concentration [16]. For the first point, freeze–thaw cycles and CaCl_2_ activation are the two main methods to lyse platelets, but CaCl_2_ activation seems to be less effective and alters the morphology of MSCs [17]. Platelet lysate concentrations also appear to be critical for the expansion of MSCs. Russel et al. [18] demonstrated that an addition of 30% or more of ePL leads to a rapid decrease in cell proliferation. The concentration of heparin added to prevent clotting of the medium is perhaps the most difficult point to control. It depends on a wide range of parameters such as the basal medium used (calcium concentration) and the number of platelets present in the lysate [19]. Two-dimensional models using platelet lysate and heparin are the most commonly investigated method to culture MSCs without FBS. But to avoid the use of heparin, research into 3D models must be carried out. Naskou et al. [20] were the first group to generate encapsulated equine bone marrow MSCs using equine platelet lysate and calcium chloride. They showed that supernatant from the 3D model showed higher concentrations of cytokines such as IL-1β, IL-10 and TGF-β and also VEGF, which is a key factor for cell multiplication, while viability seemed not to be impacted. Based on the literature, all research to replace FBS in equine MSC culture was performed with adipose-, bone marrow- or cord blood-derived MSCs. As demonstrated by Ceusters et al. [21], MSCs can also be taken directly from the muscle of the horse (mdMSCs). This is less painful and easier to obtain than through bone marrow aspiration or liposuction. Recently, in a paper currently in the submission process about muscle-derived progenitor cells (mdP-cells), our team demonstrated that these cells can be expanded in 3D models with equine plasma [22]. Moreover, the team also reported that mdP-cells were able to inhibit T-lymphocyte proliferation. However, a large number of pathologies in horses are associated with polymorphonuclears and especially with neutrophils [23,24].

Therefore, our aim is to optimize the culture of mdMSCs and avoid the use of FBS by investigating the expansion of mdMSCs using 10% platelet lysate, a richer growth factor supplement compared to equine plasma, in novel 2D and 3D approaches. Furthermore, we also focus on immunomodulatory properties of the generated mdMSCs by studying the inhibition of reactive oxygen species (ROS) and myeloperoxidase (MPO), which are both released by neutrophils and are keys players in the inflammatory process.

## 2. Material and Methods

### 2.1. Acquisition and Preparation of Equine Platelet Lysate

**Figure 1:**
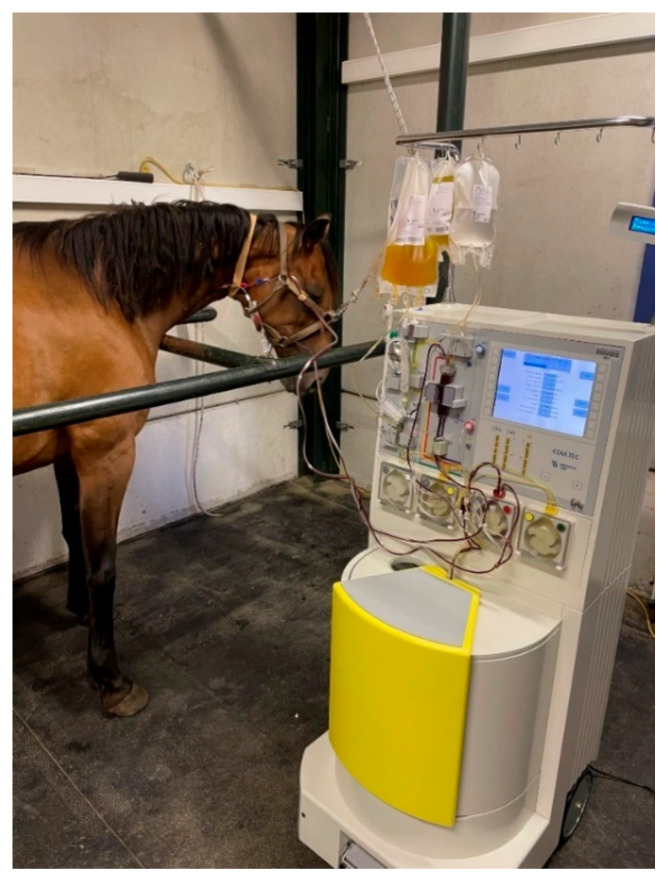
Collection of ePL from an awake horse on which apheresis is being carried out using the COM.tec plasmapheresis device.

The collection of platelet-rich plasma (PRP) was performed using the COM.tec plasmapheresis system from Fresenius Kiabi (Bad Homburg, Germany) and specifically by using the kit C5L Platelet set from the same company. The optimum parameters were set according to the manufacturer’s instructions. PRP obtention was performed on three awake equine blood donors from the European Center of Horse in Mont le Soie (Figure 1). Each platelet count from the 3 donors was calculated by plasmapheresis (Table 1). The apheresis products from the three donors were combined to create a single pool, which was then evenly distributed into Falcon 50 mL tubes (Corning, NY, USA) under laminar flow. The platelets were subsequently broken down using two freeze–thaw cycles. These cycles consisted of freezing PRP at −80 °C overnight and thawing it at 37 °C for 2 h (2×) to obtain equine platelet lysate (ePL). Based on this ePL, two different supplements were performed for the two different models. For the 3D model, there was no modification and ePL was used as is (supplement 1). For the 2D model, ePL had to be prepared differently to avoid coagulation of the whole medium. After the two freeze–thaw cycles, the generated ePL was further centrifuged at 3500× *g* for 30 min at room temperature to pellet the platelets and obtain the supernatant, called platelet-poor plasma (PPP). The entire PPP was then transferred to another Falcon 50 mL except for 5 mL in which to resuspend the platelet pellet. One of these 5 mL was added to the rest of the PPP (45 mL) to obtain a highly concentrated ePL and to form our final supplement for the 2D model (supplement 2). The two supplements were stored at −20 °C until further use. In this study, the 3 donors were screened for the most common infectious diseases (equine infectious anemia, equine viral arteritis, West Nile virus).

### 2.2. Optimization of Heparin Concentrations for 2D Mesenchymal Stromal Cell Culture with 10% Platelet Lysate

Before comparing 2D and 3D models, tests were first performed to find the optimal heparin concentration for the 2D model. 50,000 frozen mdMSCs from three horses between passage 4 to passage 6 were thawed and cultured in parallel in T-25 cm^2^ flasks with DMEM/Ham’s F12 (Gibco, Grand Island, NY, USA) complemented with 10% ePL (supplement 2) and increasing concentrations of heparin (Stemcell Technologies, Koln, Germany) (Table 3). Two parameters were used to evaluate the effect of heparin on our new medium. Macroscopic visual inspection was performed to observe putative coagulation of the medium, and microscopic inspection was conducted to evaluate the morphology and the confluence of the mdMSCs. All flasks were incubated at 37 °C with 20% O_2_ and 5% CO_2_. The entire medium was changed every two days until confluence (i.e., entire surface area was covered with a continuous layer of cells).

### 2.3. Muscle Microbiopsy for Initial Culture and Complete Process to Culture mdMSCs Using Platelet Lysate in a 2D Model

Equine muscle-derived mesenchymal stromal cell samples were obtained by microbiopsy from the triceps brachii with local anesthesia, as described by Ceusters et al. [21], and stored at 4 °C for a maximum of 72 h. This study and procedure were conducted according to the guidelines of and approved by the Animal Ethical Commission of the University of Liege (reference number 1609). Five horses were used as donors for this amplification and differentiation study (one stallion and four mares; ages ranged between 17 and 25 years). Briefly, the 15 to 20 mg microbiopsies were washed with phosphate buffer saline (PBS, Gibco, Grand Island, NY, USA), cut into small pieces, and placed in a 48-well plate. Each well thus contained one explant and 120 µL of DMEM/Ham’s F12 medium (Gibco, Grand Island, NY, USA) complemented with 10% of supplement 2 and a final concentration of 1.44 IU/mL in heparin (Stemcell Technologies, Koln, Germany). This entire medium is referred to as a 2D medium for ease of reference. When the first cells came out from the microbiopsy, 50 µL of fresh 2D medium was added every 3 to 4 days. At confluence, passage 1 was performed. At each passage, the supernatant was discarded and PBS was added to remove any trace of serum before trypsinization. The explant-derived cells were detached in each well by using 150 µL of synthetic trypsin (TrypLE express 1×, Gibco, Grand Island, NY, USA). After detachment, 300 µL of PBS was added to neutralize the TrypLE, and a centrifugation step was performed at 200 g for 10 min. mdMSCs in the pellet were then resuspended in 1 mL of HBSS (Gibco, Grand Island, NY, USA) and further isolated on a discontinuous Percoll (Gibco, Grand Island, NY, USA) gradient (3 layers: 15%, 25%, and 35%). After centrifugation at 1250× *g* for 20 min without brake, the cells between the 15 and 25% layers were collected, washed with PBS, resuspended in 5 mL of 2D medium, and placed in a T-25 cm^2^ flask. The entire medium was replaced every 2 or 3 days maximum. Passages 2 and 3 involved the same process as used in passage 1 to detach and obtain the cells. When the 6 T-175 cm^2^ flasks were confluent, mdMSCs were detached as previously described, resuspended in PBS, and counted on a Thoma cell-counting chamber. Cell viability was assessed using a trypan blue exclusion test. An amount of 40 µL of mdMSCs solution was sampled, and 10 µL of trypan blue was added. Then, the generated mdMSCs were freshly used or cryopreserved in a Cryostor^®^ CS5 solution at a concentration between 0.5 and 10 million (BioLife Solutions, Bothell, WA, USA) at −80 °C for short-term storage (up to 2 months) or in liquid nitrogen for the longer term (2 months to several years).

### 2.4. Muscle Microbiopsy for Initial Culture and Minimally Manipulated Process to Culture mdMSCs Using Platelet Lysate in a 3D Model

Equine muscle-derived mesenchymal stromal cells used for the 3D model were obtained from the same five horses as for the 2D model. The study and the procedure were conducted as described in Section 2.3. Briefly, Microbiopsies were prepared as described for the 2D culture. Each well thus contained one explant and 150 µL of DMEM/Ham’s F12 medium (Gibco, Grand Island, NY, USA) complemented with 10% of supplement 1 (as described in Section 2.1). This entire medium is referred to as a 3D medium for ease of reference. No maintenance was required because the semisolid matrix, which is constituted by multiple layers, supports cell proliferation without the addition of fresh medium. Once the cells were confluent, they were released by dissolving the gel using a proteolytic enzyme called Cell Collect 3D, developed by Revatis S.A. (WO 2021/165451 [25]). Powder of this enzyme was diluted in 20 mL of PBS and filtered on a 0.22 µM membrane. At passage 1, 100 µL of this proteolytic enzyme was added in each well. After 5 to 7 min, the plasma gel containing the cells was dissolved, and 200 µL of PBS was added to neutralize the effect of this enzyme. The cells were then harvested in a Falcon 15 mL and centrifuged at 200× *g* for 10 min before being resuspended directly into 18 mL of 3D medium and placed in a T-175 cm^2^ flask. At confluence, mdMSCs were detached following the same protocol as described before and amplified until confluence in 6 T-175 cm^2^ flasks. Finally, mdMSCs were detached and counted on a Thoma cell-counting chamber with trypan blue to assess the number of cells and the viability, as described for the 2D model. The generated mdMSCs were freshly used or cryopreserved in a Cryostor^®^ CS5 solution at a concentration between 0.5 and 10 million (BioLife Solutions, Bothell, WA, USA) at −80 °C for short-term storage (up to 2 months) or in liquid nitrogen for the longer term (2 months to several years).

### 2.5. Flow Cytometry Analysis of the Immunophenotype of the Generated Cells

Briefly, mdMSCs generated from 2D and 3D models were thawed, washed with PBS, and centrifuged at 600× *g* for 5 min. The cell pellets were then resuspended in 500 µL FACS buffer (Miltenyi Biotec, Leiden, The Netherlands), and a sample was taken for cell counting and viability assessment. The cells were then incubated with conjugated antibodies labelled with their respective fluorochromes, CD44/FITC (BioRad, Hercules, CA, USA), CD45/PerCP (BioRad, CA, USA), MHCII/PE (BioRad, Hercules, CA, USA), and unconjugated primary antibodies (CD90) (Washington State University-Monoclonal Antibody Center, WA, USA), for 15 min at 4 °C in the dark. After incubation, the cells were diluted with FACS buffer, respectively, as described in Table 2, and centrifuged at 600× *g* for 5 min. For the CD90 marker, a second antibody conjugated to FITC (Anti IgM, Abcam, Boston, MA, USA) was added to the primary unconjugated antibodies and incubated for 15 min at 4 °C in the dark. After two washes with FACS buffer, data acquisition was performed using the MACSQuant 10 analyzer (Miltenyi Biotech, Leiden, The Netherlands).

### 2.6. Trilineage Differentiation of Equine Mesenchymal Stromal Cells

MdMSCs generated with the 2D culture model were seeded at a concentration of 200,000 cells/well in a 24-well plate with complete medium including heparin. MdMSCs obtained with the 3D model were seeded in 2D with complete medium including heparin to evaluate the potency of differentiation. Upon reaching confluence for both cultures, the complete medium was replaced with StemPro^®^ Chondrogenesis, StemPro^®^ Osteogenesis, and StemPro^®^ Adipocytes Differentiation Medium for chondroblast, osteoblast, and adipocyte differentiation, respectively. For negative controls, complete medium with platelet lysate was replaced every 2–3 days. The plate was then incubated for 14 days at 37 °C in a 5% CO_2_ incubator, with the differentiation medium changed once a week according to the manufacturer’s instructions. Subsequently, the differentiated cells were fixed with 4% formaldehyde and stained with Alcian Blue, Alizarin Red, and Oil Red O for 15 min to evaluate the presence of chondroblasts (detecting mucopolysaccharides in cartilage matrix), osteoblasts (identifying calcium deposits), and adipocytes (assessing lipid content and triglycerides), respectively.

### 2.7. Specific Immunological Extraction Followed by Enzymatic Detection (SIEFED) Myeloperoxidase (MPO) Analysis

The MPO inhibitory capacity of generated mdMSCs from the 3D and 2D models was assessed using SIEFED MPO. As described by Franck et al. [26], this technique involves specific immuno-extraction of the enzyme followed by revelation of its activity. Increasing cell concentrations were used, and a known concentration of MPO (Bioptis, Liège, Belgium), the same for each condition, was also added to the wells. After 2 h of incubation at 37 degrees Celsius, the wells were washed 4 times with PBS/Tween20 buffer (0.1%) (GE Healthcare), and reagents for revealing MPO activity were added (10 µL NaNO_2_ (GE Healthcare)), 10 µL H_2_O_2_ (GE Healthcare), and 40 µL of the fluorescent probe amplex red (Fisher Scientific, Merelbeke, Belgium). The fluorescence signal at 590 nm was directly measured by Fluoroskan Ascent (Thermo Fisher Scientific, Waltham, MA, USA) during a kinetic period of 30 min. The MPO control was considered as our positive control (100% enzymatic activity).

### 2.8. Effect of mdMSCs on the ROS Production by Neutrophils

The equine neutrophils used in this study were obtained from the whole blood of five healthy horses from the Equine Clinic of the University of Liège (Liège, Belgium). Neutrophils were isolated following the method described by Pycock et al. [27] and suspended in Ringer lactate (RL) (B.Braun, Melsungen, Germany) before activation. Smears of neutrophils isolated from horses were placed on microscope slides, stained by Diff-Quik (Medion Diagnostics, Freiburg, Germany), and observed by microscope (Zeiss, Axioskop, Zaventem, Belgium). Based on 5 different microscopic fields per horse (100 cells/field), the purity of the neutrophil population was estimated, and the cell preparation consisted of ≥95% neutrophils. Neutrophils (0.5 × 10^6^ neutrophils/well) were incubated for 10 min in the wells of a white microtiter plate with varying concentrations of isolated mdMSCs in RL buffer. Dilutions of the cell suspension and reagents were made to achieve a final volume of 200 µL. Superoxide anion production was quantified by chemiluminescence (CL) according to Franck et al. [28], using L-012 (Fujifilm Wako Chemicals Europe GmbH, Neuss, Germany) as the chemiluminescent probe. Prior to measurement, 10 μL L-012 (1.2 mg/mL in distilled water) and 10 μL phorbol 12-myristate-13-acetate (PMA) (16 μM in 1% DMSO in ultrapure H_2_O) (Merck, Bornem, Belgium) were added. The CL response was monitored for 30 min immediately after the addition of PMA and expressed as the integral of the total CL emission. Two control experiments were conducted: one with PMA-activated neutrophils without mdMSCs and another with unstimulated neutrophils without mdMSCs, where PMA was replaced by its vehicle solution (1% DMSO in H_2_O).

### 2.9. Statistical Analysis

Experiments were conducted using mdMSCs from five horses and blood samples from five horses for neutrophil isolation. Each experiment was performed in duplicate. In the figures, data are presented as mean ± standard deviation (SD) and expressed as relative values compared to the positive control group, which was set at 100%. For statistical analysis, raw data were used. The mean of each technical replicate was calculated for each horse, and the means and SDs from the five independent experiments were used for the analysis (*n* = 10). MPO and ROS activities were analyzed using repeated measures ANOVA (MedCalc Software bv, 22.032 version, Ostend, Belgium). This two-factor study included repeated measures on the culture process (2D and 3D). For tests of between-subjects effects, if the *p*-value for “groups” is low (*p* < 0.05), there are significant differences between the two culture models. For tests of within-subjects effects, if the *p*-value for “Factor” is low (*p* < 0.05), there are significant differences between measurements. If the *p*-value for “Group × Factor” is low (*p* < 0.05), it indicates that the difference between measurements depends on group membership.

## 3. Results

### 3.1. Optimization of Heparin Concentrations for 2D Mesenchymal Stromal Cell Culture with 10% Platelet Lysate

**Figure 2:**
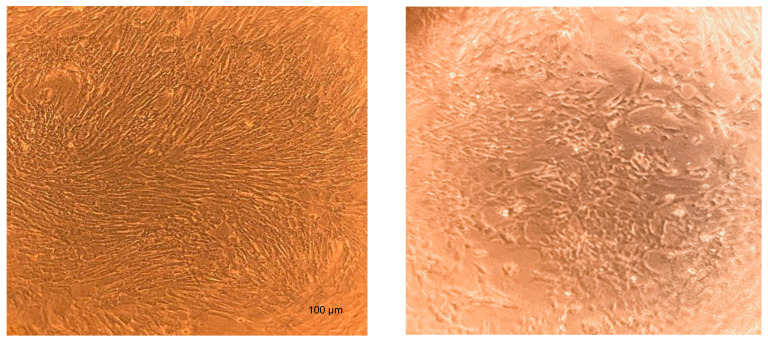
Representative microphotographs by optical microscope (×100) of mdMSCs cultured after 7 days with 10% platelet lysate and 1.44 IU/mL of heparin (**left**) and 3 IU/mL of heparin (**right**) (Horse 1).

mdMSCs cultured in 2D with 10% platelet lysate and increasing concentrations of heparin showed mixed results (Table 3). Indeed, we observed a rapid coagulation of the entire medium with the lowest heparin concentrations, 0.36 and 0.72 IU/mL. Then, an addition of 1.08 IU/mL of heparin showed the first visual anticoagulant effects. Coagulation of the whole medium occurred with cells of the first horse but not immediately with the cells of the second and third horses. It was only after the first medium change on day 2 that a thin layer of clotting appeared. From 1.44 IU/mL of heparin, no coagulation was observed even after medium change and cells proliferated in 2D (Figure 2). mdMSCS expanded under the last two conditions with 10% platelet lysate supplemented with 2.5 and 3 IU/mL heparin showed no coagulation of the entire medium but a change in morphology (Figure 2, on the right), as they tended to cluster into islets.

### 3.2. Muscle Microbiopsy for Initial Culture and Complete Process to Culture mdMSCs Using Platelet Lysate in a 2D Model

**Figure 3:**
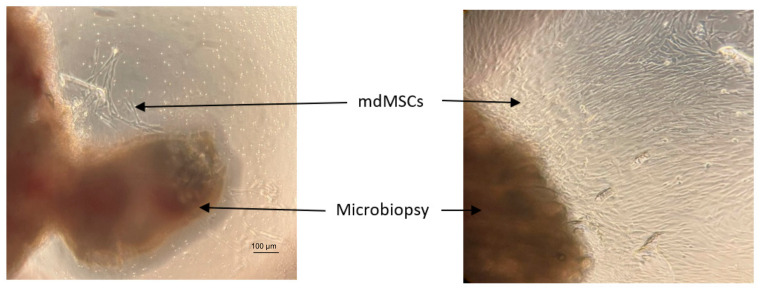
Representative microphotographs by optical microscope (×100) of mdMSCs cultured after 4 days (**left**) and 8 days (**right**) with 10% platelet lysate and 1.44 IU/mL of heparin (Horse 3).

Initially, each piece of muscle microbiopsy placed in a 48-well plate showed some cells coming out of the explant after 3 to 4 days. Then, the cells began to divide rapidly, and after 8 days we observed a cellular carpet of well-arranged cells, as shown in Figure 3.

### 3.3. Muscle Microbiopsy for Initiating Culture of mdMSCs Using Platelet Lysate in a 3D Model

**Figure 4:**
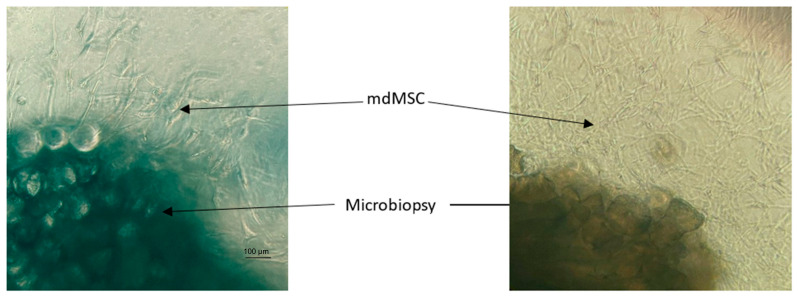
Representative microphotographs by optical microscope (×100) of mdMSCs cultured in 3D after 4 days (**left**) and 8 days (**right**) with DMEM/Ham’s F12 medium supplemented with 10% platelet lysate (Horse 3).

Cells emerged from the explant after 3 to 4 days as described for the 2D model and divided rapidly. Figure 4 showed the rapidity of cell growth after 4 to 8 days. We also observed that mdMSCs showed a morphological shift compared to the mdMSCs of the 2D model and were able to grow out through the different layers of the gel.

### 3.4. Comparison of the Total Number of Muscle-Derived Mesenchymal Stromal Cells Harvested with the 2D and 3D Models

**Figure 5:**
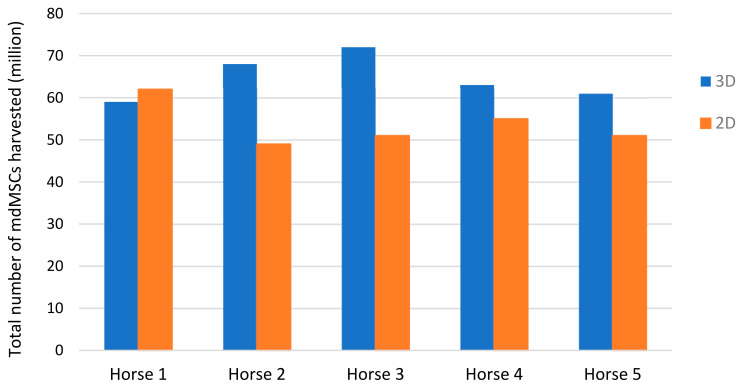
Total number of mdMSCs harvested with the 3D and 2D models at passage 3.

mdMSC of the five horses were amplified with the 2D and 3D models until confluence in six T-175cm^2^ flasks. mdMSCs that came from the 2D model were amplified during 28 ± 4 days, while those from the 3D model had a total amplification time of 22 ± 2 days. The average total number of cells generated with the 3D model was 64.6 ± 5.32 million mdMSCs as shown in Figure 5, and cell viability evaluated by the trypan blue exclusion test was 83 ± 4%. In parallel, the 2D model generated an average of 57.2 ± 7.66 million mdMSCs with a viability of 87 ± 2.6%.

### 3.5. Trilineage Differentiation and Immunophenotyping of Generated mdMSCs Obtained with 2D and 3D Models

**Figure 6:**
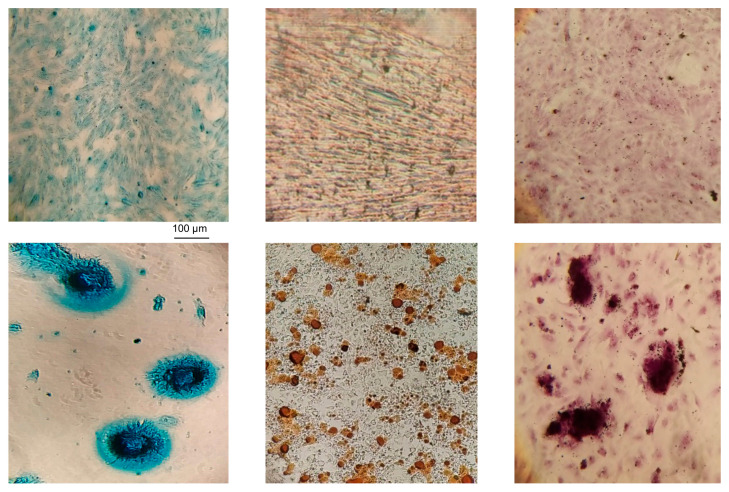
Representative microphotographs obtained by optical microscope (×100) of trilineage differentiations of mdMSCs (from the 3D model at passage 3) with, respectively, for the upper line chondroblast (**left**), adipocyte (**between**), and osteoblast (**right**) cells cultured without differentiation media. Lower line is composed of differentiated chondroblasts (**left**), adipocytes (**between**), and osteoblasts (**right**).

The mdMSCs generated in the 2D and 3D models retained their stem cell characteristics in accordance with the recommendations of the International Society for Cellular Therapy (ISCT), Table 4. They demonstrated the ability to undergo trilineage differentiation (adipogenic, osteogenic, chondrogenic), as shown in Figure 6, for the mdMSCs generated with the 3D model and exhibited plastic adherence. No difference was observed in term of differentiation for mdMSCs generated by the two models.

### 3.6. SIEFED MPO Analysis

**Figure 7:**
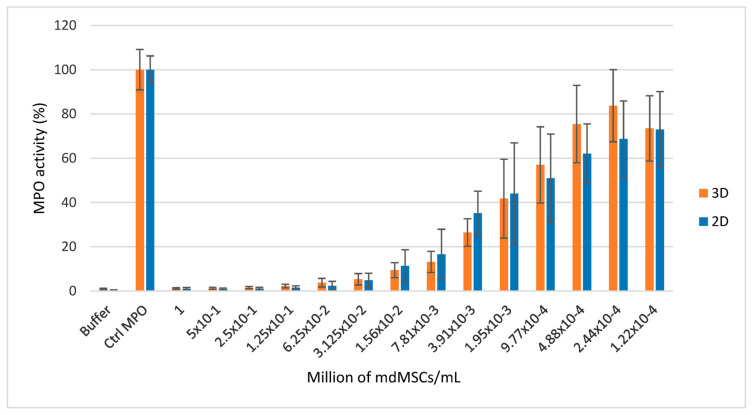
Effect of mdMSCs (passage 3) on the activity of equine MPO measured by SIEFED. Results from five independent experiments with two technical replicates for each concentration (*n* = 10). The means ± SD are shown as relative percentages compared to the MPO control, which was performed without mdMSCs and defined as 100% response.

SIEFED MPO analysis showed that mdMSCS cultured in 2D and 3D induced a strong dose-dependent inhibition of MPO activity (Figure 7). According to the statistical analysis, the dose-dependent inhibition appeared to be very significant (*** *p* < 0.001). The inhibition reached about 90% for the 15,000 cells/mL and about 50% for the 1950 cells/mL levels compared to the medium containing only MPO and set as 100% response. Moreover, the statistical analysis showed no significant difference in MPO inhibition between the 2D and 3D mdMSCs groups.

### 3.7. Effects of mdMSCs on the ROS Production by Neutrophils

**Figure 8:**
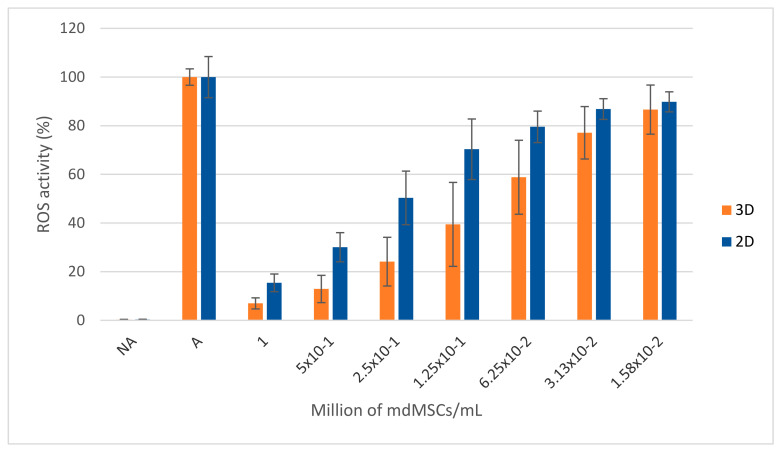
Effects of different concentrations of mdMSCs (passage 3) in Ringer lactate on the ROS production by neutrophils (*n* = 10). NA and A represent, respectively, non-activated and activated neutrophils alone. Means ± SD are shown in relative percentages. Stimulated neutrophils without mdMSCs (A) is defined as 100% response. Means ± SD are shown in relative percentages of A.

The data were obtained from five independent experiments using different neutrophil and mdMSC preparations. The stimulation of PMNs induced intense ROS production compared to non-activated PMNs (Figure 8). The addition of mdMSCs significantly inhibited this ROS production in a dose-dependent manner for all conditions (** *p*-value < 0.01). This experiment demonstrated that generated mdMSCs with the 3D model had a significantly higher inhibition on ROS release (*** *p*-value < 0.001) compared to the mdMSCs generated from the 2D model. mdMSCs from the 3D model at a concentration of 0.5 M/mL inhibited ROS activity by about 90%, while mdMSCs from the 2D model inhibited ROS activity by about 60% at the same mdMSC concentration.

## 4. Discussion

We demonstrated for the first time the feasibility of using equine platelet lysate as an alternative to fetal bovine serum for the expansion of equine muscle-derived mesenchymal stromal cells in innovative 2D and 3D cultures. We also identified the most suitable heparin concentration for the 2D model based on the PRP provided by the plasmapheresis COM.tec system. Indeed, heparin concentration seems to be a key issue for the culture of mdMSCs in 2D. Below 1.44 IU/mL of heparin, the entire medium forms a gel matrix and mdMSCs grow in a 3D gel matrix as for the 3D model. From 2.5 IU/mL of heparin, the morphology of mdMSCs tends to change with cellular clustering)and proliferation is impaired, meaning that confluence can never be achieved, as also demonstrated by Hemeda et al. [29].

In addition, the harvest of the 3D model yielded a higher average total of 64.6 million mdMSCs, while the 2D model yielded an average of 57.2 million mdMSCs. Therefore, the expansion of the mdMSCs is achievable in these two innovative culture models. However, we are aware that we cannot compare the culture time durations for two main reasons. First, the production of the two supplements is different. Indeed, to avoid coagulation, a certain part of the platelet lysate is withdrawn for the 2D medium. Secondly, a Percoll gradient was voluntarily omitted from the 3D model in order to have a minimally manipulated method. These two main factors increased the time duration for the 2D culture. Nevertheless, the higher number of cells harvested in the 3D model can be explained by the ability of mdMSCs to proliferate in multiple layers rather than in a 2D monolayer model. Thus, the surface is demultiplied, and mdMSCs have more space to obtain a high number of cells. Our results also showed that the viability of mdMSCs was lower in the 3D culture (83%) compared to the 2D culture (87%). Both models are of particular interest for autologous cell therapy such as the treatment of musculoskeletal injuries, tendonitis, and other common conditions affecting horses [30]. In fact, the ability to provide over 50 million MSCs in 20 days from a single muscle microbiopsy opens up great opportunities for equine treatments, particularly for 3D printing, which requires large numbers of cells and would eliminate any risk of rejection [31]. In addition, this is particularly advantageous for meeting the needs of equine clinical trials and commercial manufacturing of cell-based therapies for equine patients. As also demonstrated by Yaneselli et al. [32] on equine bone marrow mesenchymal stem cells (BM-MSCs), ePL provides adequate proliferation and is a suitable alternative to the use of FBS, but higher cell numbers can be achieved for the 2D culture model by using other tools such as the Hyperflask, which has the surface area of 10 T-175 cm^2^ flasks and could provide around 100 million mdMSCs if the ratio is optimized.

The differentiation capacity of equine mesenchymal stromal cells (eMSCs) can vary depending on the isolation technique and the sample site [33]. Nevertheless, as already shown by Naskou et al. [34] with equine bone marrow MSCs, we also demonstrated that the generated mdMSCs cultured with ePL retained their multipotency (osteoblast, adipocyte, and chondroblast) and their immunophenotype. mdMSCs generated from the 3D model do not express CD44, an adhesion marker. This is expected, because the cells grow throughout the various layers of the gel and do not adhere to the flask. Santos’s team also showed that cultures of eMSCs from synovial membrane lost their CD44 marker when they were encapsulated in alginate hydrogel [35].

Moreover, the proliferation rate of eMSCs cultured in 10% ePL is comparable to eMSCs cultured in FBS, which represents a significant advancement for equine cell culture [34,36,37]. This use of a blood product from the same species, as recommended by the ISCT and EMA, is suitable for the replacement of this common supplement.

The third part of this study on the immunomodulatory properties of mdMSCs showed interesting results. SIEFED’s analysis of the MPO showed that 2D- and 3D-generated mdMSCs strongly inhibited the activity of MPO with a dose-dependent curve. Franck et al. [38] already described this ROS and MPO inhibition through experiments with mdMSCs from 2D culture with FBS, while Bogers et al. [39] described the anti-inflammatory properties of BM-MSC in 3D. Our results confirmed that the mdMSCs generated with our two novel models retained their ability to inhibit MPO and ROS activities, which are key players in the inflammatory process [40,41]. However, we showed for the first time that mdMSCs from the 3D model had a statistically higher inhibitory potency of ROS production compared to mdMSCS from the 2D model.

In conclusion, this study showed that the two models are promising for equine mesenchymal stromal cell culture and cell therapy. Indeed, the replacement of FBS by ePL provides a xeno-free medium, avoiding all the disadvantages associated with FBS. In addition, the immunomodulatory properties of the generated mdMSCs can be used to fight the inflammatory process found in a wide range of pathologies. To further characterize the mdMSCs generated in our two models, evaluating cell senescence could be a valuable perspective. Indeed, cellular senescence is a critical parameter in all cell cultures, and a recent study showed that eMSCs cultured in ePL exhibited half as many chromosomal aberrations compared to those cultured in FBS [15].

## Figures and Tables

**Table 1:** Key features of plasmapheresis for platelet lysate harvesting.

Horse	Plasma Flow (mL/Min)	Collected Volume (mL)	Platelet Yield
1	36	842	8.10 × 10^11^
2	40	792	6.93 × 10^11^
3	38	816	7.42 × 10^11^
Average	38	817	7.48 × 10^11^

**Table 2:** Antibodies for analyzing the cellular proteins on mdMSCs.

Antibody Target	Clone	Dilution
CD44	MCA1082	25
CD45	MCA87	5
MHCII	MCA1085	25
CD90	DH24A	50

**Table 3:** Increasing concentrations of heparin added in the basal medium supplemented with 10% platelet lysate (coagulation (X) or not (/) of the whole medium).

Heparin Concentration (IU/mL)	Horse 1	Horse 2	Horse 3
0.36	X	X	X
0.72	X	X	X
1.08	X	X *	X *
1.44	/	/	/
2.5	/	/	/
3	/	/	/

* Coagulation after the first medium change.

**Table 4:** Immunophenotyping of muscle-derived mesenchymal stromal cells generated with the 2D and 3D models (at passage 3) for CD44, CD45, MHCII, and CD90 markers (all results are expressed as a percentage).

	Two-Dimensional Model	Three-Dimensional Model
CD44	98.50 ± 1.38	1.96 ± 0.79
CD45	0.06 ± 0.01	0.31 ± 0.13
MHCII	0.06 ± 0.07	0.26 ± 0.16
CD90	99.39 ± 0.09	98.90 ± 0.73

## Data Availability

Please contact the author for supplementary data and material. Data Availability Statements are available in section “MDPI Research Data Policies” at https://www.mdpi.com/ethics, (accessed on 23 July 2024).
